# Contrasting Mutation Rates from Specific-Locus and Long-Term Mutation-Accumulation Procedures

**DOI:** 10.1534/g3.111.001842

**Published:** 2012-04-01

**Authors:** John W. Drake

**Affiliations:** Laboratory of Molecular Genetics, National Institute of Environmental Health Sciences, Research Triangle Park, North Carolina 27709

**Keywords:** specific-locus mutation rates, genomic sequencing for mutation rates, synonymous codons, neutral mutations

## Abstract

Until recently, the two predominant ways to estimate mutation rates were the specific-locus method and the mutation-accumulation (Bateman-Mukai) method. Both involve seeding a number of parallel lines from a small, genetically uniform population, growing as long as is feasible but not so long as to allow selection to perturb mutant frequencies, and sometimes using extreme bottlenecks to facilitate the retention of deleterious mutations. In the specific-locus method, mutations are selected according to their specific phenotypes and are confirmed by sequencing. In older versions of the mutation-accumulation method, the increase in variance of a quantitative fitness trait is measured and converted into a mutation rate. More recently, a variation on the mutation-accumulation method has become possible based on phenotype-blind genomic sequencing, which might (or might not) provide improved sampling breadth, usually at the expense of sample size. In a recent study, genomic sequencing was applied to *Escherichia coli* lines propagated for 40,000 generations and passaged daily via 5,000,000 cells. To mitigate the impact of selection, the only targets employed for rate calculations were putatively neutral synonymous mutations. The mutation rate estimate was about 6-fold lower than obtained previously with a robust specific-locus method. Here I argue that purifying selection acting to shape the strong codon preferences of *E. coli* is the probable cause of the lower estimate, rather than, for instance, a lower mutation rate in nature than in the laboratory.

Microbial mutation rates can be estimated in a number of distinct ways. In the classical specific-locus method, mutants are detected by their phenotypes following limited growth (on the order of 30 generations) starting with a number of small, mutant-free populations, and the presumed target loci are then sequenced. The advantage of the method is that growth is terminated before confounders, such as selection (differential growth of mutants *vs.* parentals), can produce a substantial effect, and this and other confounders can in any case be reconstructed and measured and the mutation rate adjusted appropriately. The impacts of jackpots (high mutant frequencies resulting from particularly early appearances of the first mutation) are marginalized by adopting the median rate of the parallel cultures. With the advent of inexpensive DNA sequencing methods, highly informative specific-locus mutation rates and spectra can be constructed based upon hundreds of mutations collected under conditions that are free from selection perturbations. This has allowed base-substitution rates to be estimated from the minority of chain-termination mutations, which are detected with high efficiency because they generate stop codons and thus truncate proteins and exhibit high penetrance, and thus constitute particularly well-defined mutational targets, in contract to missense mutations; a minor disadvantage is that chain-termination mutations sample most but not all base-substitution pathways. Carefully measured basal genomic base-substitution mutation rates are now available for seven mesophilic DNA microbes, and the values cluster closely: 0.0022, 0.0025, 0.0026, 0.0030, 0.0035, 0.0038, 0.0043, mean = 0.0032, the *Escherichia coli* value being underlined (Drake 2009). Although larger values are seen in mutator mutants, substantially smaller values are not (Drake 1993), at least using well-characterized specific-locus methods.

A very different method for estimating microbial mutation rates is a version of the classical mutation-accumulation method in which a number of parallel lines are established and propagated for various times (which can correspond to tens of thousands of generations) and genomic sequencing is then performed. Because the observed ratios of non-synonymous (missense) to synonymous (putatively silent) mutations are far smaller than the 3:1 ratio expected from random base substitutions in a genome with close to 25% of each base, strong purifying selection against missense mutations is inferred, rendering such mutations useless for rate estimations. Instead, the accumulated synonymous mutations may be used as the most neutral (selection-free) set. The advantage of this method is that the entire protein-encoding portion of the genome can be sampled. A current disadvantage is that the number of retrieved mutations may be small; for instance, an *E. coli* cell with a genomic base-substitution rate of 0.0025 per generation (Drake 1991, 2009) grown for 40,000 generations would experience ≈100 base substitutions. Of these, 86 would modify protein-coding sequences and about a fourth of that set, or 21.5, would be synonymous base substitutions.

Just such an experiment was recently described (Wielgoss *et al.* 2011). The experimental material was a set of eight long-term *E. coli* strain B cultures transferred daily for many years (mostly for 40,000 generations) by growing a severely glucose-limited sample to overnight saturation at 5 × 10^7^/ml, diluting 0.1 ml into 9.9 ml of fresh medium, and regrowing. Samples saved frozen from various generations were then subjected to genomic sequencing, and candidate base substitutions were confirmed by resequencing. Lines that had gained a mutator mutation were excluded. The resulting rate was (25 synonymous mutations)/(300,000 cumulative generations)(941,000 synonymous sites) = 8.9 × 10^−11^ per base pair per generation or, for the 4,629,812-base(pair) genome, 0.00041 base-pair substitutions per genome per generation.

The genomic base-substitution rate for *E. coli* obtained by applying the specific-locus method to the *lacI* gene in strain K12 is ≈0.0025 based on a spectrum containing 24 chain-termination mutations (Drake 1991, 2009). This value is 6.1-fold higher than the rate of 0.00041 from Wielgoss *et al.* (2011), who commented in their abstract that “our estimate represents the most accurate measure of bacterial base-substitution rates available to date” and in the text that “our estimate is probably more accurate” applying “the reasonable presumption of selective neutrality or near-neutrality for most synonymous mutations.” However, it has long been known that synonymous codon usage in bacteria is often strongly biased and is subject to substantial selection, especially in *E. coli* (Sharp and Li 1987; Ochman 2003; Sharp *et al.* 2005; Hershberg and Petrov 2008). The factors that frame the selective forces may include the rate of transcription and the associated correlation between tRNA abundance and codon bias, the rate of polypeptide folding, the location of the codon within the gene, and the distance of the gene from the replication origin. Which of these are operating in the present case must remain enigmatic because little is known about selection on codon usage under these experimental growth conditions. However, it was possible from the data posted in Wielgoss *et al.* (2011) to determine the vectors of most of their mutations with respect to codon usage. Of 23 mutations, 12 were switches from codons more often used to less often used, 7 were switches between codons similarly often used, and 4 were from codons less often used to more often used (supporting information, Table S1). This pattern informs poorly about whether frequency of use was a main driver of selection on codon usage, at least in this particular set of 40,000 generations of probably highly stressful growth in a thoroughly unnatural environment during which quite a few of the populations were taken over by mutator mutants. The neutrality assumption might be tested, for example, by examining the relationship between the codon adaptation index and the mutations observed in these populations.

Even weak selection coefficients are likely to impact mutation accumulation in a set of 40,000-generation cultures passing through daily bottlenecks of 5,000,000 cells. An informative set of these selection coefficients could, with some effort, be directly assessed in reconstruction controls starting from artificial mixtures of mutant and parental genotypes. In addition, several striking examples of the impact of a synonym on the phenotype have surfaced in a recent version of the *E. coli lacZ*α mutation assay (Zhong *et al.* 2006), wherein mutants can be detected by even slight reductions from the wild-type dark-blue phage-M13 plaque color. Five synonyms that reduce plaque color are C→G at site 44, C→T at 86 and 95, C→A at 107, and C→G at 167 (M. E. Arana and T. A. Kunkel, personal communication). (Site numbers begin before the translated sequence, and codon third positions happen not to be multiples of 3.) As in the example of the 23 mutations mentioned above, the codon-usage vectors are diverse: three point downward and two point upwards. Because the mutational target used by Wielgoss *et al.* (2011) is likely to experience purifying selection during 40,000 generations, it would be appropriate to accept the *E. coli* specific-locus rate as the best current measure, and to estimate the average selection coefficient against synonymous base substitutions from the 6-fold lower mutation-accumulation rate.

Students of spontaneous mutation may find some interesting gifts in the data of Wielgoss *et al.* (2011). The authors noted that mutations from G/C to A/T (that is, from G·C to A·T or to T·A) at synonymous sites were more prevalent than mutations from A/T to G/C, as has been noted in many other organisms (*e.g.*, Lynch 2007), suggesting that still poorly defined selective forces drive A/T to G/C to achieve the observed genomic ratios. Of the 23 index mutants that could be traced back to the genomic sequence from their compilations, 16 were G/C→A/T (14 as transitions), 4 were A/T→G/C, and 3 were A·T→T·A, a strong bias for an organism with a genomic G·C content of 50.8% [GenBank:NC_012967.1] (Table S1). A similar result can be observed among 80 base substitutions in the *E. coli lacI* gene (whose 1083 protein-encoding base pairs are 56.3% G·C, significantly different from the corresponding *E. coli* value at *P* ≈ 0.01): 58 were G/C→A/T (45 as transitions), 12 were A/T→G/C, 3 were G·C→C·G, and 7 were A·T→T·A (Farabaugh 1978; Halliday and Glickman 1991) (Table S2). Thus, this mutational bias appears to be general rather than focused on synonymous mutations.

In addition to the mutated base pair itself, the data of Wielgoss *et al.* (2011) provide hints of associations with G/C base pairs at nearby sites. Of the 23 mutated sites and independently of whether that site was G/C or A/T, the numbers of G/C base pairs extending in both directions from –5 through the mutated site to +5 were 16, 10, 16, 18, 15, **16** (the mutated site), 15, 10, 13, 10, 11 compared with an expected value of 23 × 0.508 = 11.7 per site (Table S1). When the pooled numbers were compared with the expected values by a replicated goodness-of-fit test, the deviation was significant (G_P_ = 4.88, 1 df, *P* < 0.05). In addition, the numbers differed among positions (G_H_ = 14.7, 9 df, *P* = 0.0995), with a few underlined positions showing low or significant *P* values (for positions –5 and –3, *P* = 0.078; for position –2, *P* = 0.007). Combining the overall deviation from the expected incidences and the heterogeneity of the data, the total statistic was significant (G_T_ = G_P_ + G_H_ = 19.5, 10 df, *P* ≤ 0.05). Although these results are not statistically impressive because of the numbers mutations, they prompted a similar inspection of the 80 *lacI* mutations of Halliday and Glickman (1991), which yielded 40, 50, 40, 42, 61, **61**, 39, 51, 38, 33, 57 compared with an expected number of 80 × 0.563 = 45.04 per site (Table S2). When these numbers were analyzed, the statistic for the pooled data was not significant (G_P_ = 0.05, 1 df, *P* = 0.8307), but the heterogeneity statistic was highly significant (G_H_ = 39.2, 9 df, *P* < 0.0001), as was the total statistic (G_T_ = 39.2, 10 df, *P* < 0.0001). The number at –1 had *P* < 0.0001, that at +5 had *P* = 0.0081, and that at +4 (high A/T) had *P* = 0.0049. A pronounced tendency toward hypermutability within G/C-rich sequences was later noted for the DNA polymerase of coliphage RB69, a relative of phage T4 (Bebenek *et al.* 2001). The molecular bases for these tendencies can probably be best characterized by pre-steady-state kinetics and estimates of efficiencies of mismatch extension using appropriate primer·template combinations, as in Xia *et al.* (2011) and Arana *et al.* (2011).

## Supplementary Material

Supporting Information
