# Supplementary material for: Contrasting Mutation Rates from Specific-Locus and Long-Term Mutation-Accumulation Procedures
Source: G3 (Bethesda). 2012 Apr 1;2(4):483–5. doi: 10.1534/g3.111.001842 (PMC3337476; doi:10.1534/g3.111.001842)
Supplement: Supporting Information [file supp_2_4_483__index.html]

Supporting Information 

# Contrasting Mutation Rates from Specific-Locus and Long-Term Mutation-Accumulation Procedures

## Supporting Information for Drake, 2012

**Files in this Data Supplement:**

- Supporting Information - Tables S1 and S2 (PDF, 64 KB)
- Table S1 - Properties of 23 *E. coli* synonymous mutations (PDF, 56 KB)
- Table S2 - Properties of 80 *E. coli lacI* mutations (PDF, 57 KB)
