# Supplementary material for: Contrasting Mutation Rates from Specific-Locus and Long-Term Mutation-Accumulation Procedures
Source: G3 (Bethesda). 2012 Apr 1;2(4):483–5. doi: 10.1534/g3.111.001842 (PMC3337476; doi:10.1534/g3.111.001842)
Supplement: Supporting Information [file supp_2.4.483_001842SI.pdf]

Table S1 Properties of 23 *E. coli* synonymous mutations

Site	Target	Path	AA	FCU
122,591	3 T T G C G **T** G C A T G 3	T→A	arg	.38→.06
132,062	3 C T G C A **C** G A G T C 3	C→T	his	.43→.57
212,865	3 G C C A T **T** C G T A T 2	T→C	ile	.51→.42
307,594	4 G T C G G **G** C T C T C 3	G→A	gly	.15→.11
420,328	3 A T G G G **T** G C G G G 5	T→G	gly	.34→.15
756,799	3 C T G G A **C** G T A C T 2	C→T	asp	.37→.63
1,083,668	4 C T G G C **G** A G C G T 3	G→A	ala	.36→.21
1,317,194	2 A A A G G **C** G T G A T 2	C→T	gly	.40→.34
2,087,738	4 G G T C G **C** G T T G G 3	C→A	arg	.40→.06
2,095,621	3 C T G C T **G** G G C T G 4	G→A	leu	.50→.04
2,251,393	4 C C G A G **C** G G C A C 4	C→T	ser	.28→.15
2,772,320	5 C G C G C **A** A A A A C 1	A→C	ala	.21→.27
2,983,794	3 G G T G A **C** T A C A T 1	C→T	asp	.37→.63
3,061,109	4 G C C T C **G** A T T G G 2	G→A	ser	.15→.12
3,107,610	2 C G T A T **T** C T G C A 3	T→A	ile	.51→.07
3,124,208	3 A G C G T **G** A G T G A 2	G→A	val	.37→.15
3,141,566	4 C C G C T **C** A G C A T 2	C→T	leu	.10→.10
3,308,106	2 T A T G C **G** C T A A T 1	G→A	ala	.36→.21
3,407,922	5 G G C G G **G** C G C T A 3	G→T	gly	.40→.34
3,409,316	1 A A A T C **T** A C C C C 4	T→G	ser	.15→.15
4,107,018	3 A A G C C **A** C T G A C 3	A→T	pro	.19→.16
4,133,104	3 G A A C G **C** G T A G A 2	C→T	arg	.40→.38
4,313,510	4 C T G G C **G** A A A G A 1	G→A	ala	.36→.21

Target = the mutating central base (bold face) and its 5 flanking bases on either side (with the target codon underlined). Path = the mutation at the target base. FCU = the Fractional Codon Usage of the wild-type and the mutated codons (http://openwetware.org/wiki/Escherichia_coli/Codon_usage).

Table S2 Properties of 80 *E. coli lacI* mutations

Site	Path	No.	Target	Site	Path	No.	Target
023	G→C	1	G G G T G **G** T G A A T	186	C→T	4	C G T G G **C** A C A A C
031	G→A	3	A A T G T **G** A A A C C	186	C→A	3	C G T G G **C** A C A A C
042	C→T	1	A G T A A **C** G T T A T	192	A→G	1	A C A A C **A** A C T G G
054	T→C	2	C G A T G **T** C G C A G	198	C→ A	1	A C T G G **C** G G G C A
056	G→A	1	A T G T C **G** C A G A G	201	G→A	2	G G C G G **G** C A A A C
057	C→T	1	T G T C G **C** A G A G T	201	G→T	2	G G C G G **G** C A A A C
075	C→T	2	T G T C T **C** T T A T C	201	G→C	2	G G C G G **G** C A A A C
080	C→T	1	C T T A T **C** A G A C C	206	C→T	2	G C A A A **C** A G T C G
083	A→G	2	A T C A G **A** C C G T T	213	T→A	1	G T C G T **T** G C T G A
086	G→T	1	A G A C C **G** T T T C C	222	G→A	1	G A T T G **G** C G T T G
093	G→A	4	T T C C C **G** C G T G G	293	C→T	1	C C G A T **C** A A C T G
102	A→T	1	G G T G A **A** C C A G G	377	C→T	2	T C G C G **C** A A C G C
104	C→T	6	T G A A C **C** A G G C C	419	C→T	5	A T G A C **C** A G G A T
105	A→C	1	G A A C C **A** G G C C A	558	T→A	2	C G C A T **T** G G G T C
140	G→A	1	A A A A A **G** T G G A A	582	T→G	1	G C T G T **T** A G C G G
141	T→A	1	A A A A G **T** G G A A G	683	G→T	1	A A G G C **G** A C T G G
143	G→T	1	A A G T G **G** A A G C G	768	A→T	1	C A A C G **A** T C A G A
150	C→A	1	A G C G G **C** G A T G G	777	C→A	2	G A T G G **C** G C T G G
158	G→T	1	T G G C G **G** A G C T G	842	G→A	1	T A G T G **G** G A T A C
168	A→G	1	G A A T T **A** C A T T C	845	T→G	1	T G G G A **T** A C G A C
183	T→A	1	C C G C G **T** G G C A C	885	T→G	1	G C C G T **T** A A C C A
185	G→A	7	G C G T G **G** C A C A A	926	A→C	2	A A A C C **A** G C G T G

The data are from Halliday and Glickman (1991), whose sites are numbered according to Farabaugh (1978); the mutation at site 192 has been corrected to A→G. "No." indicates the number of mutants of that kind at that site.
