# Supplementary material for: Contrasting Mutation Rates from Specific-Locus and Long-Term Mutation-Accumulation Procedures
Source: G3 (Bethesda). 2012 Apr 1;2(4):483–5. doi: 10.1534/g3.111.001842 (PMC3337476; doi:10.1534/g3.111.001842)
Supplement: Supporting Information [file supp_2.4.483_TableS1.pdf]

Table S1 Properties of 23 *E. coli* synonymous mutations

Site	Target	Path	AA	FCU
122,591	3 T T G C G **T** G C A T G 3	T→A	arg	.38→.06
132,062	3 C T G C A **C** G A G T C 3	C→T	his	.43→.57
212,865	3 G C C A T **T** C G T A T 2	T→C	ile	.51→.42
307,594	4 G T C G G **G** C T C T C 3	G→A	gly	.15→.11
420,328	3 A T G G G **T** G C G G G 5	T→G	gly	.34→.15
756,799	3 C T G G A **C** G T A C T 2	C→T	asp	.37→.63
1,083,668	4 C T G G C **G** A G C G T 3	G→A	ala	.36→.21
1,317,194	2 A A A G G **C** G T G A T 2	C→T	gly	.40→.34
2,087,738	4 G G T C G **C** G T T G G 3	C→A	arg	.40→.06
2,095,621	3 C T G C T **G** G G C T G 4	G→A	leu	.50→.04
2,251,393	4 C C G A G **C** G G C A C 4	C→T	ser	.28→.15
2,772,320	5 C G C G C **A** A A A A C 1	A→C	ala	.21→.27
2,983,794	3 G G T G A **C** T A C A T 1	C→T	asp	.37→.63
3,061,109	4 G C C T C **G** A T T G G 2	G→A	ser	.15→.12
3,107,610	2 C G T A T **T** C T G C A 3	T→A	ile	.51→.07
3,124,208	3 A G C G T **G** A G T G A 2	G→A	val	.37→.15
3,141,566	4 C C G C T **C** A G C A T 2	C→T	leu	.10→.10
3,308,106	2 T A T G C **G** C T A A T 1	G→A	ala	.36→.21
3,407,922	5 G G C G G **G** C G C T A 3	G→T	gly	.40→.34
3,409,316	1 A A A T C **T** A C C C C 4	T→G	ser	.15→.15
4,107,018	3 A A G C C **A** C T G A C 3	A→T	pro	.19→.16
4,133,104	3 G A A C G **C** G T A G A 2	C→T	arg	.40→.38
4,313,510	4 C T G G C **G** A A A G A 1	G→A	ala	.36→.21

Target = the mutating central base (bold face) and its 5 flanking bases on either side (with the target codon underlined). Path = the mutation at the target base. FCU = the Fractional Codon Usage of the wild-type and the mutated codons (http://openwetware.org/wiki/Escherichia_coli/Codon_usage).
